# The more, the better: influence of family planning discussions during the maternal, newborn and child health continuum of care on postpartum contraceptive uptake and method type among young women in Ethiopia

**DOI:** 10.12688/gatesopenres.14626.1

**Published:** 2023-05-09

**Authors:** Lisa M. Calhoun, Jennifer Winston, Lenka Beňová, Ilene S. Speizer, Thérèse Delvaux, Solomon Shiferaw, Assefa Seme, Celia Karp, Linnea Zimmerman, Thomas van den Akker

**Affiliations:** 1 Carolina Population Center, The University of North Carolina at Chapel Hill, Chapel Hill, North Carolina, 27516, USA; 2 Athena Institute, Vrije Universiteit Amsterdam, Amsterdam, North Holland, The Netherlands; 3 Department of Public Health, Institute of Tropical Medicine, Antwerp, Belgium; 4 Department of Maternal and Child Health, The University of North Carolina at Chapel Hill, Chapel Hill, North Carolina, USA; 5 School of Public Health, Addis Ababa University, Addis Ababa, Addis Ababa, Ethiopia; 6 Department of Population Family and Reproductive Health, Johns Hopkins Bloomberg School of Public Health, Baltimore, Maryland, USA; 7 Department of Obstetrics and Gynecology, Universiteit Leiden Medical Center, Leiden, South Holland, The Netherlands

**Keywords:** youth, adolescent, contraception, prenatal care, postpartum, continuum of care, integration, family planning, counseling

## Abstract

**Background: **This study examines the association between family planning (FP) discussions with health professionals during contact points on the maternal, newborn and child health continuum of care and timing of modern contraceptive uptake and method type in the one-year following childbirth in six regions of Ethiopia among adolescent girls and young women (AGYW).

**Methods**: This paper uses panel data of women aged 15-24 who were interviewed during pregnancy and the postpartum period between 2019-2021 as part of the PMA Ethiopia survey (n=652).

**Results**: Despite the majority of pregnant and postpartum AGYW attending antenatal care (ANC), giving birth in a health facility, and attending vaccination visits, one-third or less of those who received the service reported discussion of FP at any of these visits. When considering the cumulative effect of discussions of FP at ANC, pre-discharge after childbirth, postnatal care and vaccination visits, we found that discussion of FP at a greater number of visits resulted in increased uptake of modern contraception by one-year postpartum. A greater number of FP discussions was associated with higher long-acting reversible contraceptive use relative to non-use and relative to short-acting method use.

**Conclusions**: Despite high attendance, there are missed opportunities to discuss FP when AGYW access care.

## Introduction

Postpartum family planning (PPFP) helps to prevent unintended pregnancies following childbirth, contributes to the healthy spacing of births, and reduces unmet need for family planning ^1^. Yet, an analysis of Demographic and Health Survey (DHS) data from 21 low- and middle-income countries found that 61% of women had an unmet need for family planning (FP) within 24 months of childbirth ^2^.

The World Health Organization (WHO) recommends that women receive information about family planning (FP) throughout the maternal, newborn and child health continuum of care (referred to in this paper as the ‘continuum of care’) - during antenatal care (ANC), immediately after childbirth, during postpartum care and as part of the expanded program on immunization visits - as a cost-effective strategy to support voluntary contraceptive use and support women in achieving their ideal pregnancy intervals and family size ^3– 5^. However, the evidence about when, where and how much counseling about FP is needed during the continuum of care is not clear. Across a variety of contexts, the evidence supporting any relationship between provision of FP information at individual visit types along the continuum of care and postpartum contraceptive uptake is varied, including FP information at ANC visits ^6– 13^, at the time of childbirth ^6, 14, 15^ and in the one-year period postpartum, both within health facilities and through community-based workers ^11, 12, 16– 18^. Because some women may express the intention to use postpartum contraception during the antenatal period ^19^ but are unable to act upon that intention until after childbirth, repeat FP counseling sessions – both antenatally and postnatally – may be critical to increasing the ability of women to translate these intentions into PPFP use ^20^.

Estimates of PPFP use in Ethiopia, the site of this study, vary. A meta-analysis undertaken by Mehare and colleagues in 2020 based on 18 studies from Ethiopia that were published between 2015 and 2019 found that the pooled prevalence of PPFP use within one-year postpartum was 48.1% (95% CI: 37.0, 59.3) ^13^. However, data from the nationally representative 2016 Ethiopia DHS suggests that PPFP use by one-year postpartum was lower at 26% ^21^. A recent analysis of the 2016 DHS found that 28.2% of 15–24 year olds were using a modern FP method at one-year postpartum ^22^.

While postpartum FP use among adolescent girls and young women (AGYW) is similar to women of all ages, prior studies have demonstrated that there are numerous gaps to access to FP and maternal and newborn health services from AGYW ^23^. Globally, complications from pregnancy and childbirth are the leading causes of death among AGYW ^24^, and AGYW who experience closely spaced births are at risk of negative health outcomes for themselves and their children ^25^. Additionally, evidence from Uganda and Nigeria indicates that AGYW who experience a birth have been shown to be at greater risk of another birth within 12 months, which has negative implications for women’s educational attainment, household wealth and fertility ^26, 27^. AGYW face unique challenges to PPFP access and use related to social norms around demonstrating fecundability and community approval of FP use among young people ^28– 32^, lack of agency to use FP ^33^, limited information about contraceptive options ^34^, and provider bias whereby providers limit access to FP based on parity, marital status or age ^35, 36^. These challenges, coupled with evidence that AGYW are less likely to breastfeed exclusively to six months, which in turn reduces protection against pregnancy from LAM use and exposes them to a faster return to fertility ^37, 38^, underscores the importance of identifying relevant strategies to meet AGYW needs. Strategies such as home visits and community engagement have been identified as important approaches to increase PPFP use among first time mothers ^39^, yet there is limited evidence about the role of receipt of information about FP during the continuum of care on contraceptive uptake and method choice among AGYW in the postpartum period. Given the significant needs of AGYW in the postpartum period, it is critical that these inequities are addressed so that AGYW are assured access to high quality reproductive health services during the postpartum period.

To address these gaps, we examine the association between discussion of FP with health professionals during various contact points on the continuum of care and contraceptive use in the first year following childbirth in six regions of Ethiopia among AGYW. Specifically, we examine the association between discussion of FP during ANC, pre-discharge after childbirth, at postnatal visits within six weeks of childbirth and at vaccination visits with timing of modern contraceptive uptake and method type, including long-acting reversible contraceptive methods (LARC).

## Methods

### Ethics

At baseline, verbal informed consent to participate was obtained from a member of the household for the household questionnaire. Also, women ages 15–49 provided verbal informed consent to participate in the baseline study as well as the panel study. According to the National Research Ethics Review Guidelines in Ethiopia, women aged 15–17 are able to consent for themselves, and so parental permission was not required for their participation ^40^. Additionally, based on guidance from the National Research Ethics Review Guidelines and the IRB on record for primary data collection, written consent is not required in areas of low literacy or for studies that do not include invasive procedures such as collection of biomedical samples ^40^. At the time of each follow-up visit, the full informed consent was not administered, but respondents were asked if they had any questions and if they still agreed to participate in the study. This study and data collection procedures were approved by the AAU (Ref: AAUMF 01-008) and JHBSPH Institutional Review Boards (FWA00000287). This secondary analysis study of deidentified data was assessed by the University of North Carolina Institutional Review Board and determined exempt from further review (Study #22-1781).

### Data

This analysis utilizes a subsample from panel data collected from Ethiopian women aged 15–49 who were interviewed during pregnancy and the postpartum period between 2019–2021 as part of the Performance Monitoring for Action (PMA) Ethiopia survey. The survey was undertaken in six regions – Addis Ababa, Afar, Amhara, Oromia, Southern Nations, Nationalities and People’s Region (SNNPR), and Tigray – which represent approximately 90% of Ethiopia’s population ^40^.

In order to identify a representative sample of pregnant women, a sample of enumeration areas (EAs) was drawn with probability proportional to size by urban and rural strata. With the exception of Afar and Addis Ababa, EAs were selected separately from urban and rural strata within each region. EAs were selected from Afar and Addis Ababa without stratification because Addis Ababa does not contain rural areas and Afar is predominantly rural. The number of EAs selected per region ranged from 13 in Afar to 52 in Oromia. In September-December 2019, a census was conducted in the 217 selected EAs across the six regions. In total, 32,614 households were listed. A brief household questionnaire was administered, including a household roster. The roster was utilized to identify all women aged 15–49 for participation in the study. Women were identified for possible participation in the baseline of the panel survey if they were pregnant or had given birth within the nine weeks prior to the survey.

Follow-up surveys were administered at six-weeks, six-months, and one-year postpartum. Women were eligible for the panel if they were regular members of the household, including women who were at their parental home and intended to stay throughout childbirth and the postpartum period. During the baseline interview, an approximate gestational age and due date were calculated for pregnant women. Using this information, resident enumerators (RE) periodically contacted respondents by phone to determine when the birth occurred. Once childbirth was confirmed, RE scheduled the six-week postpartum visit. For respondents who had already given birth at the time of the baseline survey, they completed the six-week survey at the same time as the baseline survey. The six-month and one-year postpartum survey interviews were scheduled after completion of the six-week postpartum survey visit.

The baseline and follow-up interviews were conducted by RE. The baseline interview included questions about sociodemographic characteristics, previous use of any contraception, ANC attendance, discussion of FP at ANC, and gestational age. The questionnaire at six weeks included questions about childbirth, breastfeeding, discussion of FP immediately postpartum or in the peripartum period, and other questions about postpartum experiences. The questionnaire at six months included questions on the health of the child, breastfeeding, sexual activity, return to menses, contraceptive use (including a contraceptive calendar), postnatal care, childhood vaccination visits, well baby visits, and sick visits; questions about discussion of FP were asked for each visit type. The questionnaire at one-year included questions on the health of the child, breastfeeding, sexual activity, return to menses, contraceptive use (including a contraceptive calendar), postnatal care, childhood vaccination visits, and well check visits for the mother or baby; questions about discussion of FP were asked for each visit type. A description of the survey tools and the key indicators captured have been presented previously ^40^.

### Population

In total, 2,868 women who were pregnant or recently gave birth completed the baseline survey, of whom 989 were between the ages of 15 and 24 years at first interview and eligible for this study on postpartum FP use among AGYW. A total of 683 respondents ages 15–24 years completed the one-year interview (response rate = 69.1%). Our analytic sample was restricted to postpartum women ages 15–24 at the time of the baseline survey who had complete baseline, six-month, and one-year interviews (n=652, response rate = 65.9%). We excluded three women from our analysis sample who had missing information on marital status and/or parity. Twenty-eight respondents who were lost-to-follow-up at the six-week survey, but had complete baseline, six-month and one-year surveys, were retained in the analytic sample given our focus on the six-month and one-year surveys. Altogether, 87 respondents were between five and nine weeks postpartum at the time of the baseline survey, thus baseline and six-week surveys were completed at the same time.

### Measures

***Dependent variables*.** This analysis has two dependent variables: timing of modern contraceptive method initiation in the one-year postpartum period and contraceptive method type (long-acting reversible contraceptive method (LARC), short-acting methods, non-use/traditional method use) at one-year postpartum.

The first dependent variable, timing of modern contraceptive method initiation in the one-year postpartum period, was generated from retrospective contraceptive calendar data collected in the six-month and one-year surveys. At the six-month survey, data about method use, if any, and reason for discontinuation were asked for each month since birth. At the one-year survey, monthly data were asked for each month since the prior survey. Because six-month surveys were administered in a data collection window, there were not always exactly six months of calendar data available at each survey round. When compiling the data into a single yearly calendar, we used data from the six-month calendar for all available months. We used data from the one-year calendar for all months after the six-month calendar ended until one-year postpartum. Among respondents who adopted a method within the one-year period, we categorized the method as modern (coded as 1) if a respondent reported use of: female sterilization, implants, intrauterine contraceptive device (IUD), injectables, oral pills, emergency contraception (EC), male condoms, and standard days/cycle beads. For respondents who reported switching from one method to another, their month of adoption was the first month of adopting any modern method.

The dependent variable on contraceptive method type was assessed by asking respondents if they were using a method of family planning at the time of the one-year survey, and if so, what method they were using. The LARC category included female sterilization (n = 1), implants, and IUD. Short-acting methods included injectables, oral pills, EC, male condoms, and standard days/cycle beads. Non-use/traditional methods, also referred to as non-users of modern methods, included rhythm method, withdrawal, and no method. At the time of the one-year survey, seven respondents reported using LAM, but because they were not able to fulfill the definitional requirements for LAM (exclusively breastfeeding, amenorrhea, and less than six months postpartum) at one-year postpartum, they were coded as non-users of a modern method. Of the 317 respondents who were coded as non-users of a modern method, 304 were non-users, seven were LAM users who did not meet the LAM requirements, two rhythm users and four withdrawal users; therefore, it was not possible to create a separate category for traditional method use. Notably, the LARC category is primarily comprised of implant users, and the short-acting methods category primarily of injectable users. Women who reported using more than one contraceptive method were coded based on the most effective method. Respondents who reported being pregnant or were unsure of their pregnancy status (n=11) were coded as non-users of modern contraception.

***Independent variables*.** Our independent variables included four measures of women’s contact with health providers and the health system throughout the continuum of care. Table 1 presents the survey questions by survey timing. Key variables capture discussion of FP with a provider at four visit types: 1) antenatal care, 2) pre-discharge after childbirth, 3) postnatal care by six-weeks postpartum, and 4) routine childhood vaccination. For each variable, three response options were created to capture: visit attendance with no discussion of FP (coded zero), discussion of FP at the visit (coded 1), and no visit attendance (coded 2). Each variable is described in greater detail below.

**Table 1.** Survey questions from the panel study to construct independent variables.

Service	Survey questions	Survey
Antenatal care	- At any point in your pregnancy, did you see a health extension worker for antenatal care? - Did you see a professional healthcare provider, other than an HEW, for antenatal care during this pregnancy? - During your antenatal care visit, did your provider talk with you about postpartum family planning?	- Baseline survey - Six-week survey
Immediate PP	- Where did you give birth? - Before you left the facility after delivery, did a provider talk with you about using a family planning method?	- Six-week survey
Postnatal care visit by six-weeks postpartum	- Has any health extension worker visited you since delivery? - Did you go visit another professional healthcare provider other than an HEW since delivery, either for yourself or for the baby? - At your visit after delivery (either by a HEW or other professional healthcare provider) did the provider discuss family planning?	- Six-week survey
Vaccination	- Did [baby NAME] get any vaccinations? - Looking at the vaccine card, does [NAME] have? (list of vaccinations) - Copy date from the card for each vaccine that the child has - Did you receive any family planning information, referrals or services during any of the immunization visits for your baby?	- Six-month survey - One-year survey

The antenatal care variable was created by using two questions that asked about ANC visits with a health extension worker or a professional healthcare provider. Respondents who indicated they had completed any ANC visit were asked if the provider talked with them about PPFP. Respondents with missing data about ANC visit attendance were coded as “no ANC visit” (n=5). Respondents with discrepant reporting between the baseline and six-week surveys regarding discussion of FP at an ANC visit were coded as having discussed FP.

The pre-discharge FP discussion variable was constructed using questions from the six-week survey which asked about the location of childbirth and discussion of FP before being discharged from the facility after childbirth. First, respondents were asked where they gave birth and those who gave birth at a facility (government hospital, government health center, government health post, NGO/faith-based health facility, or private hospital/clinic) were asked whether a provider talked with them about using an FP method after childbirth before leaving the facility. Respondents who gave birth at their own home, another home, or “other” location were categorized as “no visit attendance” (coded 2).

For the postnatal care (PNC) variable, women were asked at the six-week survey if they were visited by a health extension worker or if they visited a professional healthcare provider for themselves or the baby since childbirth. Respondents who answered no to both questions were coded as “no visit attendance” (coded 2), while those who answered yes to either question were asked if the provider discussed FP at that visit. Respondents who reported discussing FP at either visit were categorized accordingly (“discussed FP at PNC visit” [coded 1]).

The FP at childhood vaccination variable was created by confirming if the respondent’s child received any vaccinations (verified by child vaccination cards), and, if so, whether she had received FP information at any vaccination visit in the last six months. Respondents were coded as receiving FP information at a vaccination visit if they answered ‘yes’ in the six-month or one-year survey, ‘no FP information’ if they answered that they did not receive FP information at both six-month and one-year surveys, and ‘no visit’ if the respondent indicated that the baby had not received any vaccinations at either visit. Due to concerns about FP uptake preceding the vaccination visit, we utilized vaccination date information from the vaccination card in order to calculate if the vaccination visit occurred prior to contraceptive uptake. For the creation of these variables, we utilized the first date of vaccination visit and compared that to the date of FP uptake. Only 13 respondents adopted a method prior to their first vaccine visit, and of these, 8 did not receive FP counseling at the vaccination visit. Creating a separate category for these 13 respondents did not change model results for the other categories and led to uninterpretable results for that category due to small numbers. To be consistent with the other exposure variables, we ignored the timing of vaccine visit for this analysis.

Finally, a cumulative counseling continuous variable was created to explore the combined effect of discussion of FP at visits along the continuum of care on contraceptive uptake and method type. To create the continuous variable, each of the four services were coded as ‘1’ if the respondent discussed FP at the visit and ‘0’ otherwise. The four services were summed, resulting in a continuous variable that ranged from 0 (no discussion on FP at any visit) to 4 (discussed FP at all four visit types).

### Analytic methods

***Descriptive analyses*.** Descriptive statistics were used to understand sample characteristics and differences between exposure to FP information at various visits along the continuum of care and modern method use and method type among AGYW in the postpartum period. A Sankey diagram was constructed to illustrate contraceptive uptake, method switching, and discontinuation between the six-month and one-year surveys. The descriptive analyses were weighted using one-year sample weights which adjust for the clustered survey design and for loss-to-follow-up between the baseline and follow-up surveys.

***Regression analyses*.** First, we modeled timing of modern contraceptive method initiation in the one-year postpartum period using discrete time hazard regression models. We present five separate models reflecting each type of service visit (ANC, pre-discharge, PNC, and vaccination), and the continuous variable. These models used the monthly calendar data to estimate the association between exposure variables and the time to adoption of a modern contraceptive method. Because the sample was limited to AGYW who completed both the six-month and the one-year surveys, all uncensored observations were censored at month 13. Hazard ratios were estimated in unadjusted models and also in models adjusting for sociodemographic characteristics. Sociodemographic variables were included based on previous evidence of the relationship between the covariates and contraceptive use. The hazard model adjusts for: age in years (continuous) at the baseline survey; number of live births at baseline prior to index birth (continuous); marital status at baseline (married, not married); woman’s education level (none, primary, secondary, technical & vocational, higher); religion (Orthodox Christian, other Christian, Muslim, other); and region (Tigray/Afar, Amhara, Oromiya, SNNPR, Addis). Tigray and Afar were collapsed into one region due to small sample sizes in these regions. We additionally adjusted for household wealth quintile (lowest quintile, lower quintile, middle quintile, higher quintile, highest quintile) which was created using principal component analysis based on a list of household assets and was included in the publicly available dataset. All models adjust for infant death of the index pregnancy (n=21). The hazard models additionally adjust for two time-varying variables: resumption of sexual activity and resumption of menstruation. For both variables, a respondent is coded ‘1’ in the month where the event resumed as well as subsequent months and ‘0’ for all months prior to the event taking place.

Second, we constructed multinomial logistic regression models to explore the relationship between discussion of FP with a provider along the continuum of care and contraceptive method type among AGYW at one-year postpartum. For ease of interpretation, we present relative risk ratios (RRR). The models are run with two different reference groups so that we are able to explore all comparisons for the outcome variable. We present unadjusted models and multivariate models adjusted for sociodemographic characteristics. The multinomial logistic regression models adjust for if the respondent’s menstrual cycle has returned (yes/no), but do not adjust for sexual activity because of small cell sizes which limit statistical power. At the time of the one-year survey, there were 35 respondents who had not resumed sexual activity and three who did not respond to the question. All 38 of these respondents were coded as having not resumed sexual activity and only one of them was using contraception at the one-year survey resulting in unstable estimates.

Multivariate analyses were run with Huber-White-adjusted standard errors to account for the clustering in the sample at the EA level and adjust for demographic characteristics. All analyses were performed using Stata version 17.

## Results

Table 2 presents the sociodemographic characteristics of pregnant or recently postpartum AGYW in Ethiopia who have complete baseline, six-month and one-year interviews in the PMA Ethiopia panel study in 2019/2020 (n=652). Respondents were an average age of 20.73 years (SD=0.13) and had approximately one living child at time of the baseline survey. The majority of respondents had either no formal education (23.7%) or had reached primary level (56.2%). There was a mix of religions with 34.4% being Orthodox Christian, 26.5% other Christian, 37.0% Muslim, and 2.1% other religions. Ninety-seven percent of respondents reported being currently married/in union. About 50% of the respondents came from the Oromiya region. At the time of the one-year survey, about 94% of AGYW had resumed sexual activity and about half of respondents said their menstrual cycle had resumed. Twenty-one women, or 3.4% of the sample, experienced death of an infant from the index pregnancy.

**Table 2.** Sociodemographic characteristics of women ages 15–24 who had a birth in 2019/2020 in Ethiopia and were followed postpartum for one year.

Characteristics	Weighted mean (standard deviation)	Unweighted N (Total = 652)
**Age in years at baseline**	20.73 (0.13)	652
**Number of live births at baseline prior to index birth**	0.98 (0.06)	652
	Weighted Percent	Unweighted N (Total = 652)
**Household wealth (baseline)**		
Lowest quintile	19.9	122
Lower quintile	21.5	107
Middle quintile	17.4	94
Higher quintile	22.2	137
Highest quintile	19.0	192
**Highest level of school attended (baseline)**		
None	23.7	161
Primary	56.2	328
Secondary	14.7	118
Higher	2.8	23
Technical and vocational	2.5	22
**Religion (baseline)**		
Orthodox Christian	34.4	243
Other Christian	26.5	161
Muslim	37.0	238
Other	2.1	10
**Marital/in union status (baseline)**		
Married/in union	97.0	634
Not married/in union	3.0	18
**Region (baseline)**		
Tigray/Afar	4.5	117
Amhara	19.3	114
Oromiya	50.7	203
SNNPR	22.2	159
Addis	3.2	59
**Resumed sexual activity within one year of index birth**		
Yes	93.8	614
No	6.2	38
**Menstrual cycle returned within one year of index birth**		
Yes	51.1	368
No	48.9	284
**Infant death of index birth within one-year of childbirth**		
Yes	3.4	21
No	96.6	631
**Contraceptive method use at one-year postpartum**		
Long-acting reversible method	13.8	103
Short-acting method	35.7	232
Non-use/traditional method	50.5	317

**Figure 1.**
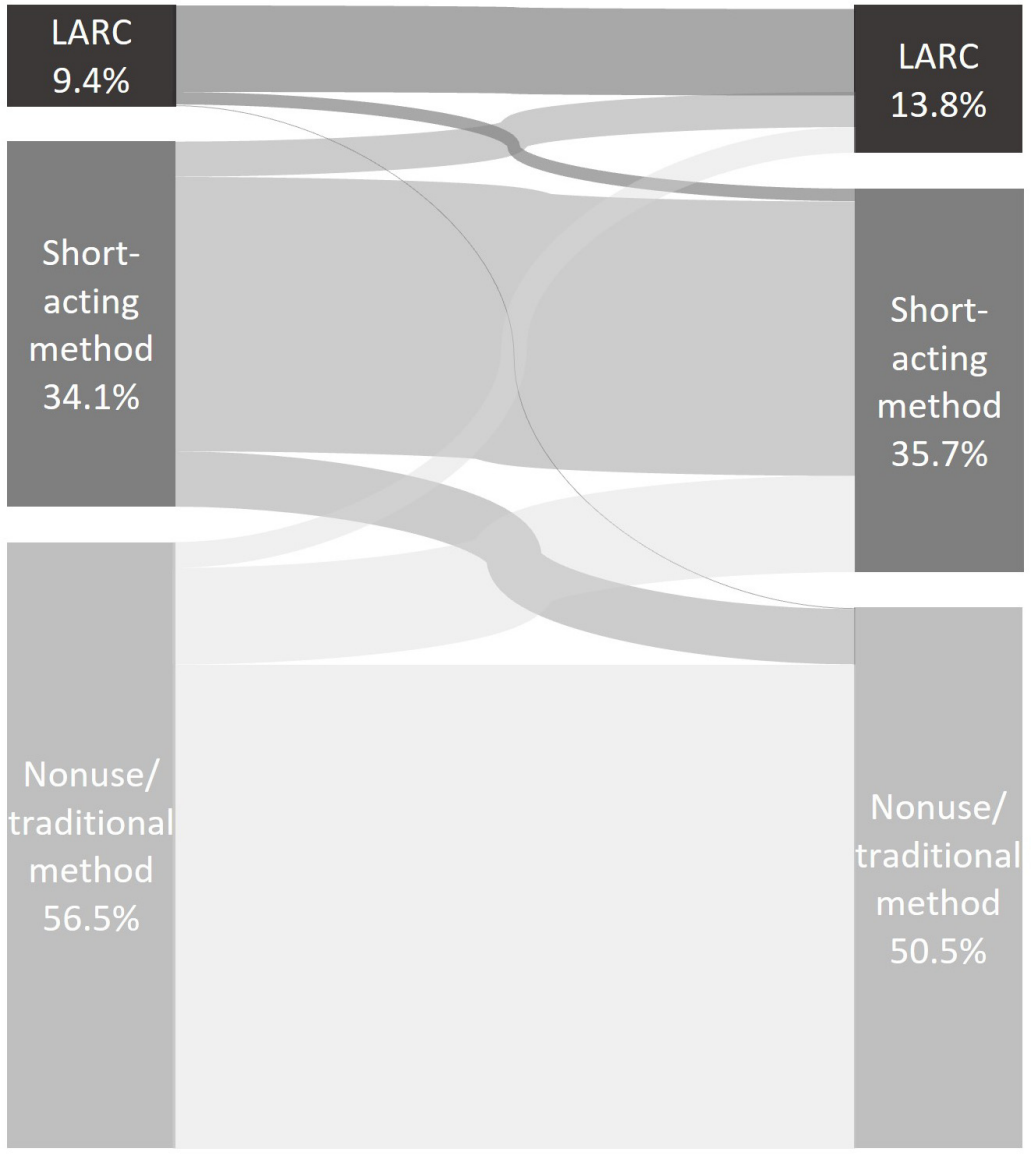
Percentage of all postpartum women ages 15–24 years reporting contraceptive use at six-months and one-year postpartum in Ethiopia (n=652).

Modern contraceptive method use was 43.5% at six-months postpartum and 49.5% at one-year postpartum. As shown in Figure 1, at six-months postpartum, about 9% of respondents reported using a LARC, 34% were using a short-acting method, and the majority of women (57%) were either not using a method or using a traditional method. At one-year postpartum, about 14% of women reported using a LARC, 36% were using a short-acting method, and about 50% were either not using a method or using a traditional method. The figure also shows method switching between the six-month and one-year surveys. Out of all respondents, 8% reported use of a LARC at six-months and were still using a LARC at one-year. A small number of respondents transitioned from using a LARC at six-months to using a short-acting method at one-year (1.2%) and even fewer discontinued use of a modern method altogether in the same period (0.1%). Of all respondents, 26% used a short-acting method at six-months and continued using it at one-year. Some respondents who were using a short-acting method at six-months transitioned to a LARC at one-year (3%). A small percentage of respondents who used a short-acting method at six-months stopped using modern methods at one-year (5%). Nine percent of respondents were non-users of modern methods at six-months and started using a short-acting method by one-year and 2% transitioned from non-use of a modern method to using a LARC. The greatest percentage of all respondents were non-users of modern methods at both six-months and one-year (45%).

Table 3 presents the percentage of AGYW who attended each type of visit and discussion of FP at each visit among those who attended the visit. The percentage of respondents who attended visits ranged by type of visit: 81% attended at least one ANC visit, 56% had an institutional birth, 30% attended an PNC visit with a health professional by six-weeks, and 86% attended a vaccination visit by one-year postpartum. Less than one third of women attending each separate visit reported discussing FP; this ranged from 17% during ANC visits to 35% at vaccination visits.

**Table 3.** Attendance at visits along the continuum of care among women ages 15–24 years who had a birth in 2019/2020 in Ethiopia (n=652).

		Discussion of FP at any visit among those who attended the visit (row %)	
Variable	Visit attendance (col %)	Yes, discussed FP	Attended visit but did not discuss FP
Any ANC visit with a health professional			
Yes	81.0	17.3	82.7
No	19.0	--	--
Had an institutional birth			
Yes	56.5	26.6	73.4
No	43.5	--	--
PNC visit with a health professional by six-weeks			
Yes	30.0	24.1	75.9
No	70.0	--	--
Any vaccination visit by one-year postpartum			
Yes	86.1	34.8	65.3
No	13.9	--	--
Total percent	100.00		

Note, all percentages are weighted using one-year sample weights and all n’s are unweighted. ANC – antenatal care; PNC – postnatal care; FP – family planning.

**Figure 2.**
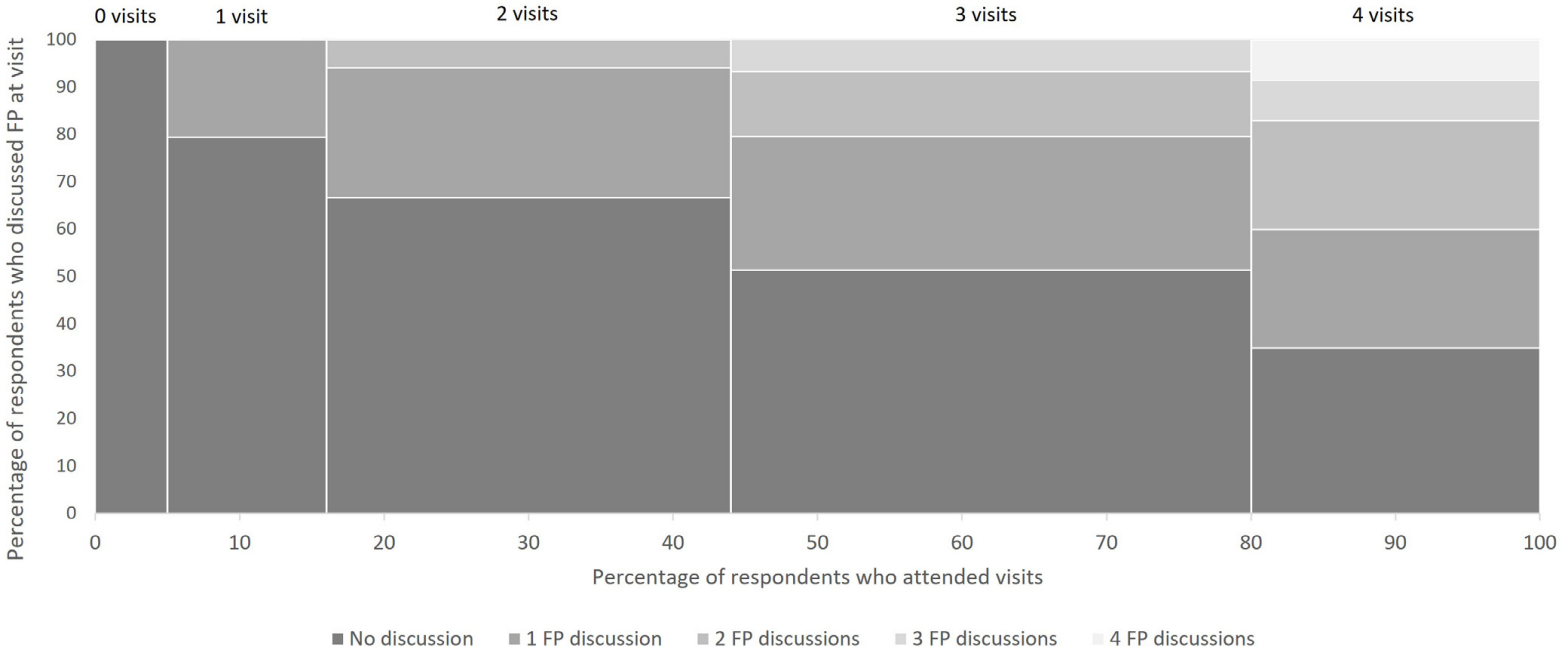
Percentage of respondents who discussed FP by number of visits attended among women ages 15–24 who had a birth in 2019/2020 in Ethiopia.

Figure 2 presents the percentage of respondents who discussed FP by number of visits attended (from none to all four). The x-axis presents the percentage of respondents who attended each visit with the width of each bar representing the percentage; overall, 5% of respondents attended no visits, 11% attended one visit, 28% attended 2 visits, 36% attended 3 visits and 19% attended all four visit types. The general pattern shows that attendance at a greater number of visits related to a higher percentage of respondents that had at least one conversation about FP. About 20% of respondents who attended one visit reported discussion of FP at that visit. Twenty-seven percent of respondents who attended two visits discussed FP at one visit and about 6% discussed at both visits. About half of those who attended three visits discussed FP at one or more visits; specifically, 28% discussed at one visit, 14% at two visits and 7% at all three visits. Among respondents who attended all four visit types, about one-third did not discuss FP at any of the four visit types, one-quarter discussed at only one of four or two of four, and about 9% discussed at either three of four or all four visit types.

Table 4 presents the bivariate association between discussion of FP with a provider at each visit along the continuum of care and contraceptive use and method type among AGYW at one-year postpartum. Nearly 60% of respondents had a value of zero for the cumulative counseling continuous variable, meaning that they did not discuss FP at any of the four service visits: ANC, pre-discharge after childbirth, PNC or at a vaccination visit. Across the number of visits, the results indicate that the higher the number of visits during which FP was discussed, the greater the reported use of a modern method. Similarly, the higher the number of visits during which FP was discussed, the higher the reported use of a LARC at one-year postpartum as compared to those who attended the visit but did not discuss FP or those who did not attend the visit. Additionally, a greater percentage of respondents who attended the service visits but did not discuss FP used a LARC as compared to those who did not attend. A similar pattern was observed for short-acting methods, but the difference in reported contraceptive use between those who discussed FP at visits and those who attended but did not discuss FP was smaller than for LARC use. In general, results from the cumulative counseling variable shows that AGYW who discussed FP at one or more visits were more likely to use a modern FP method, and those who discussed at two or more visits were more likely to use a LARC or short-acting method than be a non-user/traditional method user.

**Table 4.** Discussion of FP by contraceptive method type at one-year postpartum among women ages 15–24 who had a birth in 2019/2020 in Ethiopia.

Variable	Total (col %)	Use of any modern method (row %)	LARC	Short-acting methods	Non-use/ traditional method	Total N (unweighted)
			Row %			
Discussed FP with health professionals at an ANC visit						
Yes, discussed FP	14.0	62.5	18.5	44.0	37.5	91
Had an ANC visit but did not discuss FP	67.0	50.1	13.5	36.6	49.8	404
Did not have ANC visit	19.0	37.6	11.2	26.5	62.4	157
Total	100.0					652
Discussed FP pre-discharge after childbirth with a health professional						
Yes, discussed FP	15.0	62.4	22.4	40.0	37.6	105
Did not discuss FP	41.5	58.9	14.6	44.3	41.1	278
Did not have an institutional birth	43.5	36.1	10.0	26.1	63.9	269
Total	100.0					652
Discussed FP at PNC visit with a health professional by six-weeks						
Yes, discussed FP	7.2	74.2	29.1	45.1	25.8	50
Did not discuss FP	22.8	55.0	11.4	43.6	45.0	148
Did not attend PNC visit	70.0	45.2	13.0	32.2	54.9	454
Total	100.0					652
Received FP info at a vaccination visit by one-year postpartum						
Yes, received FP info	30.0	68.7	23.3	45.5	31.3	198
Did not receive FP info	56.1	44.7	10.9	33.8	55.3	345
Did not have a vaccination visit by one-year postpartum	13.9	27.5	4.8	22.7	72.5	109
Total	100.0					652
Cumulative counseling variable (number of visits at which FP was discussed)						
0	58.1	38.7	7.8	30.9	61.3	371
1	25.1	62.0	20.5	41.5	38.0	164
2	11.0	67.1	25.2	41.9	32.9	80
3	4.1	61.8	23.1	38.7	38.2	28
4	1.7	90.6	22.0	68.6	9.4	9
Total	100.0					652

Note, all percentages are weighted using one-year sample weights and all n’s are unweighted. ANC – antenatal care; PNC – postnatal care; FP – family planning; LARC – long acting reversible contraceptive

Table 5 presents the hazard ratios for uptake of a modern contraceptive method by one-year postpartum among women ages 15–24 in Ethiopia. First, we present the hazard ratios from unadjusted models for each exposure variable and then the adjusted model results. In all of the unadjusted models for all services along the continuum of care, the hazard of modern contraceptive uptake was significantly higher among respondents who discussed FP at a service visit relative to respondents who attended the visit but did not discuss FP. For all services, AGYW who did not attend the service visit had significantly lower hazards of modern contraceptive use as compared to women who attended the visit but did not discuss FP with a provider. In the model adjusting for sociodemographic characteristics, the hazard of modern contraceptive uptake was significantly higher among AGYW who received FP information at an ANC visit relative to those who attended ANC but did not receive FP information at the visit (HR =1.41, p≤0.05). The hazard was also significantly higher for AGYW who received FP information at a child vaccination visit by one year postpartum (HR=1.56, p≤0.001). AGYW who did not give birth at a health facility had lower hazards of modern contraceptive uptake compared to women who gave birth at a facility but did not receive FP information before leaving the facility (HR=0.67, SE=0.10, p≤0.01). In the adjusted model for every one unit increase in the cumulative counseling variable, there was a 1.34 increased hazard of contraceptive uptake within one year of childbirth. 

**Table 5.** Hazard ratio for modern FP initiation during the one-year postpartum period among women.

	Unadjusted (n=652)	Adjusted models (n=652)
Variables	Hazard ratio (SE)	Hazard ratio (SE)
MODEL 1		
Discussed FP with health professionals at an ANC visit		
Yes, discussed FP	1.45 (0.20) **	1.41 (0.20) *
Had an ANC visit but did not discuss FP (ref)	--	--
Did not have ANC visit	0.54 (0.08) ***	0.87 (0.13)
MODEL 2		
Discussed FP pre-discharge after childbirth with a health professional		
Yes, discussed FP	1.35 (0.18) *	1.25 (0.17)
Did not discuss FP (ref)	--	--
Did not have an institutional birth	0.36 (0.05) ***	0.67 (0.10) **
MODEL 3		
Discussed FP at PNC visit with a health professional by six-weeks		
Yes, discussed FP	1.50 (0.28) *	1.28 (0.25)
Did not discuss FP (ref)	--	--
Did not attend PNC visit	0.62 (0.07) ***	0.91 (0.11)
MODEL 4		
Received FP info at a vaccination visit by one-year postpartum		
Yes, received FP info	1.70 (0.23) ***	1.56 (0.21) ***
Did not receive FP info (ref)	--	--
Did not have a vaccination visit by one-year postpartum	0.47 (0.06) ***	0.79 (0.05)
MODEL 5		
Cumulative counseling variable (0 to 4 FP discussions)	1.56 (0.07) ***	1.34 (0.07) ***

ages 15–24 who had a birth in 2019/2020 in Ethiopia *p≤0.05, **p≤0.01, ***p≤0.001 Note: Adjusted models control for age of the respondent (years), number of live births at baseline, religion, region, marital status, highest level of school attended, wealth quintile, infant death of the index pregnancy, resumption of sexual activity and resumption of menstrual cycle. ANC – antenatal care; PNC – postnatal care; FP – family planning.

Table 6 presents the multivariate multinomial logistic regression results of the association between discussion of FP with a provider at multiple points along the continuum of care and contraceptive method type at one-year postpartum. After adjusting for sociodemographic characteristics, AGYW who discussed FP with a provider at ANC were significantly more likely to use a LARC than be a non-user of modern methods relative to those who attended ANC but did not discuss FP (RRR=2.38, p≤0.05). AGYW who did not attend any ANC visits were significantly less likely to use short-acting methods than to be a non-user of modern methods compared to those who attended ANC and did not discuss FP (RRR=0.51, p≤0.05). AGYW who discussed FP prior to discharge after giving birth at a health facility were more likely to use a LARC than be a non-users of a modern method (RRR=2.04, p≤0.05) and also more likely to use a LARC than a short-acting method (RRR=2.03, p≤0.05), compared to those who gave birth at a health facility but did not discuss FP prior to discharge. In contrast, AGYW who did not have an institutional birth were significantly less likely to use a short-acting method than be a non-user as compared to those who had an institutional birth but did not discuss FP prior to discharge (RRR=0.52, p≤0.05). Young women who discussed FP at PNC were significantly more likely to be a LARC user than short-acting method user as compared to those who attended PNC but did not discuss FP (RRR=3.90, p p≤0.01). AGYW who reported receipt of FP information at vaccination visits were significantly more likely to use a LARC or short-acting method than to be a non-user as compared to those who attended a vaccination visit but did not receive FP information (RRR=4.60, p≤0.001; RRR=2.84, p≤0.001, respectively). Finally, in the adjusted model with the cumulative counseling variable, there was a positive and significant relationship between greater discussion of FP and LARC use relative to non-use, short-acting use relative to non-use and LARC use relative to short-acting method use. For example, in the adjusted model, for every one unit increase in the cumulative counseling variable, there was a 2.25 higher relative risk of being a LARC user than a non-user.

**Table 6.** Multinomial regression results for the association between exposure to FP information along the continuum of care and contraceptive method type at one-year postpartum among women ages 15–24 who had a birth in 2019/2020 in Ethiopia.

Exposure variables	Unadjusted models (n=652)			Adjusted models (n=652)		
	LARC vs Non-use/ traditional	Short-acting vs Non-use/ traditional	LARC vs short- acting	LARC vs Non-use/ traditional	Short-acting vs Non-use/ traditional	LARC vs short-acting
	RRR (SE)	RRR (SE)	RRR (SE)	RRR (SE)	RRR (SE)	RRR (SE)
MODEL 1						
Discussed FP with health professionals at an ANC visit						
Yes, discussed FP	2.17 (0.82) *	1.29 (0.42)	1.68 (0.54)	2.38 (1.03) *	1.30 (0.48)	1.83 (0.65)
Had an ANC visit but did not discuss FP (ref)	--	--	--	--	--	--
Did not have ANC visit	0.39 (0.15) *	0.36 (0.12) **	1.08 (0.37)	0.52 (0.19)	0.51 (0.16) *	1.03 (0.38)
MODEL 2						
Discussed FP pre-discharge after childbirth with a health professional						
Yes, discussed FP	2.21 (0.71) *	1.14 (0.33)	1.94 (0.53) *	2.04 (0.74) *	1.01 (0.33)	2.03 (0.58) *
Did not discuss FP (ref)	--	--	--	--	--	--
Did not have an institutional birth	0.23 (0.08) ***	0.22 (0.06) ***	1.03 (0.31)	0.60 (0.24)	0.52 (0.15) *	1.17 (0.43)
MODEL 3						
Discussed FP at PNC visit with a health professional by six-weeks						
Yes, discussed FP	3.50 (1.52) **	1.37 (0.61)	2.55 (1.07) *	2.85 (1.55)	0.73 (0.39)	3.90 (1.72) **
Did not discuss FP (ref)	--	--	--	--	--	--
Did not attend PNC visit	0.57 (0.18)	0.48 (0.11) ***	1.20 (0.34)	1.00 (0.34)	0.77 (0.20)	1.31 (0.39)
MODEL 4						
Received FP info at a vaccination visit by one-year postpartum						
Yes, received FP info	4.48 (1.33) ***	2.73 (0.70) ***	1.64 (0.39) *	4.60 (1.48) ***	2.84 (0.79) ***	1.62 (0.41)
Did not receive FP info (ref)	--	--	--	--	--	--
Did not have a vaccination visit by one-year postpartum	0.18 (0.10) **	0.28 (0.12) **	0.65 (0.37)	0.43 (0.28)	0.80 (0.33)	0.54 (0.34)
MODEL 5						
Cumulative counseling variable (0 to 4 FP discussions)	2.74 (0.48) ***	1.91 (0.30) ***	1.43 (0.17) **	2.25 (0.38) ***	1.47 (0.22) **	1.53 (0.20) ***

*p≤0.05, **p≤0.01, ***p≤0.001; Note: Adjusted models control for age of the respondent (years), number of live births at baseline, religion, region, marital status, highest level of school attended, wealth quintile, infant death of the index pregnancy and resumption of menstrual cycle. ANC – antenatal care; PNC – postnatal care; FP – family planning; LARC – long acting reversible contraceptive

## Discussion

This study focuses on AGYW who had a pregnancy and are followed for one year postpartum to examine the association between discussion of FP along the continuum of care and postpartum contraceptive use. A focus on AGYW is pertinent as they often face barriers to access and use of contraception (overall or postpartum) related to social norms around marriage and childbearing ^30– 34^, lack of agency ^35^, lack of knowledge ^36^, or provider biases ^37, 38^. Further, AGYW are at risk of negative health outcomes for themselves and their children as a result rapid repeat pregnancies ^25– 27^.

In this study of recently pregnant AGYW, we found that discussion of FP at two visit types – ANC and at vaccination visits – was associated with greater uptake of a modern method of FP within one-year postpartum among young women ages 15–24 in Ethiopia. AGYW who did not give birth at a health facility had lower hazards of contraceptive uptake as compared to AGYW who did, regardless of whether FP was discussed or not. When considering the cumulative effect of discussing FP at the four visit types, we found that discussion of FP at a greater number of visits was associated with an increased hazard of uptake of modern contraception by one year postpartum. Additionally, AGYW who discussed FP at more visits were more likely to use a LARC or a short-acting method relative to non-use.

Despite the evidence of the effectiveness of counseling, use of services was low across most services, with approximately half of women giving birth in a facility (56%) and fewer receiving a PNC visit by six-weeks postpartum (30%), and only one-quarter of those who attended these two visit types reported discussing FP with a provider. Nearly 9 in 10 women reported attending a child vaccination visit and more than one-third of those attending discussed FP with a health professional, resulting in 30% of all respondents in the sample reporting having received FP information during a vaccination visit within one-year. Overall, only 19% of respondents reported attending all four visit types and 9% of those attending discussed FP with a health professional at all four visits, resulting in only about 2% of all respondents in the sample reporting receiving information at all four visits. Despite more than 80% of AGYW attending at least one ANC and child vaccination visit, discussion of FP at these visits was low, particularly at ANC. These missed opportunities for discussing FP are important, as a higher percentage of women who discussed FP at these visits used any modern method within one-year of childbirth and were also more likely to use a LARC by one-year postpartum. 

Regarding discussion at ANC, our results differ from two studies undertaken in Ethiopia which did not find a significant relationship between receipt of PPFP counseling at ANC and contraceptive uptake by six months among women of all ages ^12, 41^. Our results that focus on AGYW may differ from these earlier studies because ANC visits might be the young women’s first foray into the healthcare system and thus provision of FP information during ANC visits may be more important for this less experienced group. Inconsistent with other studies, we did not find that discussion of FP prior to discharge after childbirth in a health facility was associated with uptake of a modern method ^11, 12, 14, 15^. This distinction from earlier studies might reflect the fact that a greater percentage of first births take place in facilities and we found that having a facility birth was associated with uptake, whether or not information was specifically provided. We also found that discussion of FP at vaccination visits was associated with uptake of a modern method, but no association was found between discussion of FP at a postnatal care visit by six-weeks and FP uptake. Many studies do not differentiate between postnatal care visits and child vaccination visits, but among those that do, our findings are consistent for vaccination visits ^11, 12, 17, 41^. For AGYW, and all women, discussion of FP at child vaccination visits is considered acceptable as the young person has already begun childbearing and may have unmet family planning needs. Given that routine immunization requires multiple visits over the first nine months of a child’s life, these visits may provide timely opportunities to deliver information about FP to AGYW in the postpartum period ^17^.

Our analysis highlights the role of discussion of FP with health professionals – especially when repeated more than once - and use of LARCs. We found that AGYW who discussed FP at ANC, pre-discharge and at vaccination visits were more likely to use a LARC than be a non-user. Additionally, AGYW who reported a FP discussion pre-discharge and at PNC visits by six weeks were more likely to use a LARC than a short-acting method. Use of FP, including more effective methods, such as LARCs, among AGYW in the postpartum period will help to avoid rapid repeat pregnancies among these young women. Exposure to FP information at multiple visits along the continuum of care among AGYW may provide young women with more information about FP options and give them a greater understanding of what method they can use at each point in time in the postpartum period ^42^. This information is critical, as some methods can be used immediately following childbirth, such as IUDs, whereas initiation of others must be delayed. That said, there is also a risk providers may engage in biased or directive counseling as a result of policies and practices that incentivize provision of specific methods, including LARCs ^43^. Ultimately, counseling related to PPFP should center on AGYW’s preferences, needs, and circumstances to support them in achieving their reproductive goals following childbirth.

Our results are similar to findings from Cleland and colleagues (2015) who showed that repeat counseling sessions antenatally and postnatally lead to increased PPFP uptake among women of all ages ^20^. Furthermore, it is increasingly apparent from studies of women of all ages that FP counseling should take place during both ANC and PNC, and not just postnatally, in order to support women in achieving their reproductive goals through voluntary PPFP use ^41^. This may be in part because women often determine their postpartum contraceptive intentions during the antenatal period ^19^. We found that for the AGYW in this study, the need for repeat counseling, both at ANC and after childbirth, is particularly relevant for the uptake of LARCs which is consistent with findings from a multi-country study on immediate uptake of postpartum IUD among women of all ages ^44^.

Our results differ from other studies in several ways, which may explain any differences observed. First, our exposure variable compared those who discussed FP at the visit to respondents who attended the visit but did not discuss FP and those who did not attend, rather than anyone who did not discuss FP at the visit. Most other studies did not differentiate between respondents who attended but did not discuss FP and those who did not attend visits at all. We found that attendance alone, without a FP discussion, was associated with increased uptake of FP. This might be due to recall (FP info was provided but women did not remember), residual confounding (attendance of services, especially with such low uptake of some of them such as childbirth and six-week PNC visits, is linked to socio-economic empowerment which is also related to ability to obtain FP), or might be true and reflect exposures to FP information and provision opportunities beyond the discussion with a provider (e.g. posters in the health facility, discussion with peers in the waiting room, opportunity to obtain FP during the same visit to a health facility, etc.).

Our study has several limitations. First, our measures of discussion of FP at different visit types do not allow for the determination of the exact number of times FP was discussed, the duration and factual content of these exchanges, or an understanding of whether the AGYW or the health worker initiated this discussion. For example, an AGYW may have had multiple ANC visits, but we were only able to determine if she discussed FP at any one of these visits. This limitation also applies to PNC and vaccination visits. AGYW were asked if at these visits if FP was discussed or talked about, but no information was available on depth on what topics were covered nor on what was understood by the respondent. With the outcome that explores method type at one year postpartum, there is the chance the AGYW started a method and then stopped before one-year. There are a small number of women in our sample who became pregnant within the one-year follow-up period and additionally, there may be respondents who were trying to get pregnant again, in which cases non-use of modern contraception is appropriate. These respondents may have discussed FP at visits and are non-users, which may bias the results to the null value. Additionally, our study is subject to recall bias; this is reduced because of the prospective nature of the study design, but women may still not be able to accurately recall what was discussed at earlier visits. Finally, we are unable to differentiate early and older adolescents given the limited sample size of AGYW in this secondary dataset.

There are a number of strengths to this study as well. We followed women prospectively from pregnancy to one-year postpartum thus allowing us to capture information closer to visit dates. There was limited loss-to-follow-up in this study since PMA used resident enumerators to help with identification and follow-up. Though this study does not capture all regions in Ethiopia, it does include a geographic area covering 90% of the population, thus suggesting the results are generalizable to most of the country. Finally, a key difference with our study is that our sample is limited to AGYW and few studies have focused only on AGYW. Barriers to PPFP use in this population are critical to understand due to the health consequences of closely spaced births and increased complications from pregnancy and childbirth experienced by AGYW ^24– 26^. AGYW face unique barriers to accessing FP, and our study highlights that discussion of FP throughout the continuum of care results in higher uptake of a modern method within one-year postpartum and also use of LARCs.

## Conclusions

Ethiopia has made considerable gains in supporting AGYW to achieve their reproductive goals through voluntary use of modern contraception, including in the postpartum period over the last two decades ^11, 38, 45^. This has been due in part to progressive government policies and programs which improved access to FP services among AGYW and supported women’s empowerment through education and reduction in child marriage, amongst other efforts ^38, 45^. In a context of limited use of institutional birth and postnatal care visits, our findings demonstrate that increased discussion of FP with health providers during the continuum of care results in increased modern FP use among AGYW in the one-year postpartum period.

Yet, there are several missed opportunities for discussion of FP, as less than one in five AGYW reported discussing FP at two or more visits. Improved training of health professionals to counsel or provide information on FP to AGYW across the continuum of care is needed to promote free choice about contraceptive use, method type and timing. These trainings will help ensure that FP discussions actually take place along the continuum of care. Events that promote and provide information on PPFP should be undertaken at a range of other entry points such as schools or youth centers in order to reach young women who do not come to health facilities or meet with health extension workers. Future research should focus on assessing receipt of FP information at each contact point, including each ANC or immunization visit, to explore if more contacts during the antenatal or postnatal period result in increased FP uptake among AGYW. Improving integration of FP with health visits along the continuum of care will enable young women to avoid rapid and repeat unintended pregnancies and select and use the contraceptive method of their choice. 

## Data Availability

The quantitative survey data with de-identified household/female datasets for the Ethiopia panel study, and questionnaires, are available via PMA Datalab for research purposes; registration and a description of the proposed research or analysis is required.
