# Supplementary material for: The more, the better: influence of family planning discussions during the maternal, newborn and child health continuum of care on postpartum contraceptive uptake and method type among young women in Ethiopia
Source: Gates Open Res. 2023 May 9;7:67. [Version 1] doi: 10.12688/gatesopenres.14626.1 (PMC10323130; doi:10.12688/gatesopenres.14626.1)
Supplement: Peer review history

# Peer review: Reviewer response for version 1

**DOI:** 10.21956/gatesopenres.15944.r33708

**Published:** 2023-07-05

**Authors:** James N. Kiarie

**Affiliations:** 1 Department of Sexual and Reproductive Health and Research, World Health Organization, Geneva, Switzerland

This is a well reported study that uses panel data of women aged 15-24 who were interviewed during pregnancy and the postpartum periods. However the timing of data collection on outcomes and exposures overlap in two important ways. Exposure to information during vaccination visits continued over 9 months postpartum. Some women provided baseline data including exposure to antenatal counseling during their 6 week postpartum visit. This raises a serious concern of recall bias since users maybe more likely to report exposure than non users.

 The authors should:

1. Include this limitation in their discussion of results;
2. consider a sensitivity analysis excluding the 87 respondents were between five and nine weeks postpartum when they provided information on antenatal exposure to information;
3. discuss their findings that reported discussion of FP highest at vaccination visits

Is the work clearly and accurately presented and does it cite the current literature?

Yes

If applicable, is the statistical analysis and its interpretation appropriate?

Yes

Are all the source data underlying the results available to ensure full reproducibility?

Yes

Is the study design appropriate and is the work technically sound?

Yes

Are the conclusions drawn adequately supported by the results?

Partly

Are sufficient details of methods and analysis provided to allow replication by others?

Yes

Reviewer Expertise:

Contraception, infertility, epidemiology, health systems, HIV and STIs

I confirm that I have read this submission and believe that I have an appropriate level of expertise to confirm that it is of an acceptable scientific standard.

# Peer review: Reviewer response for version 1

**DOI:** 10.21956/gatesopenres.15944.r33225

**Published:** 2023-06-12

**Authors:** Iqbal H. Shah

**Affiliations:** 1 Department of Global Health and Population, Harvard T.H. Chan School of Public Health, Boston, MA, USA

A well-written paper. Suggestions and comments for clarifications or additional information are:

1. Provide the health system context, including who is tasked to provide FP information, the content of such information/discussion.
2. Define what is meant by "provider". What cadre of health professionals are included and if the association of FP discussion and FP uptake varies by type of provider (as found in some studies). 
3. Add the Consort diagram for analysis sample.
4. Female sterilization is not part of LARCs. There is only one case which could have been excluded from the analysis sample or the category renamed as LARCs and Permanent methods.
5. The aim is to examine the impact of FP discussion during the continuum of care, but results show both of having had the visit and having had discussion on FP during the visit. Those who did not visit were technically not exposed to receiving the information at the visit. The objectives should be restated or the analysis and results to be divided into two parts - visiting or not visiting; and among those visited who had discussed FP and who did not. Tables 5 and 6 should be based on only those who have had the visit. The Cumulative Counselling Visit 0 (zero) seems to include both who had a visit but did not discuss FP and those who did not visit (had home delivery, for example). Conceptually, these are different groups with the former addressing the objective of the paper. 
6. The multivariate analysis adjust for marital status with highly skewed distribution of 97% being married at baseline. Inclusion of this variable should be reconsidered.
7. Further analysis of 5% who were using at 6 month, but stopped at 12 months can be insightful in terms of reasons, % counselled and, if yes, by whom.

Is the work clearly and accurately presented and does it cite the current literature?

Yes

If applicable, is the statistical analysis and its interpretation appropriate?

Partly

Are all the source data underlying the results available to ensure full reproducibility?

Yes

Is the study design appropriate and is the work technically sound?

Partly

Are the conclusions drawn adequately supported by the results?

Yes

Are sufficient details of methods and analysis provided to allow replication by others?

Yes

Reviewer Expertise:

Reproductive health

I confirm that I have read this submission and believe that I have an appropriate level of expertise to confirm that it is of an acceptable scientific standard.
